# The Omentum in Obesity-Associated Cancer: A Hindrance to Effective Natural Killer Cell Migration towards Tumour Which Can Be Overcome by CX3CR1 Antagonism

**DOI:** 10.3390/cancers14010064

**Published:** 2021-12-23

**Authors:** Eimear Mylod, Fiona O’Connell, Noel E. Donlon, Christine Butler, John V. Reynolds, Joanne Lysaght, Melissa J. Conroy

**Affiliations:** 1 Cancer Immunology and Immunotherapy Group, Department of Surgery, Trinity Translational Medicine Institute, Trinity College Dublin, St James’s Hospital, Dublin 8, Ireland; mylode@tcd.ie (E.M.); donlonn@tcd.ie (N.E.D.); jlysaght@tcd.ie (J.L.); 2 Department of Surgery, Trinity St. James’s Cancer Institute, Trinity College Dublin, St James’s Hospital, Dublin 8, Ireland; oconnefi@tcd.ie (F.O.); chbutler@tcd.ie (C.B.); 3 Gastro-Intestinal Medicine and Surgery, St. James’s Hospital, Dublin 8, Ireland; reynoldsjv@stjames.ie; 4 Cancer Immunology Research Group, Department of Physiology, School of Medicine, Trinity College Dublin, Dublin 2, Ireland

**Keywords:** chemokines, natural killer cell, oesophageal cancer, fractalkine, IL-15

## Abstract

**Simple Summary:**

Oesophagogastric adenocarcinomas (OAC) are cancers of the food pipe and stomach which have a strong link with obesity. Natural killer (NK) cells are assassins of the immune system and are crucial for eliminating cancer. We have shown previously that NK cells are pulled into fat in OAC patients by a signalling protein called fractalkine (CX3CL1). Once in fat, NK cells die or are profoundly altered. This diminishes their ability to kill the tumour. We report that exposure to fat can reduce movement of NK cells towards the tumour. However, if a drug called a CX3CR1 antagonist is used to antagonise the receptor for fractalkine, we can restore NK cell movement towards the tumour. When we activate NK cells with a protein called IL-15, fractalkine can reduce its effect on NK cells. This provides further evidence for using CX3CR1 antagonists to reduce NK cell migration to fat and boost NK cell movement to the tumour.

**Abstract:**

Oesophagogastric adenocarcinomas (OAC) are obesity-associated malignancies, underpinned by severe immune dysregulation. We have previously shown that natural killer (NK) cells preferentially migrate to OAC omentum, where they undergo phenotypic and functional alterations and apoptosis. Furthermore, we have identified the CX3CR1:fractalkine (CX3CL1) pathway as pivotal in their recruitment to omentum. Here, we elucidate whether exposure to the soluble microenvironment of OAC omentum, and in particular fractalkine and IL-15 affects NK cell homing capacity towards oesophageal tumour. Our data uncover diminished NK cell migration towards OAC tumour tissue conditioned media (TCM) following exposure to omental adipose tissue conditioned media (ACM) and reveal that this migration can be rescued with CX3CR1 antagonist E6130. Furthermore, we show that fractalkine has opposing effects on NK cell migration towards TCM, when used alone or in combination with IL-15 and uncover its inhibitory effects on IL-15-mediated stimulation of death receptor ligand expression. Interestingly, treatment with fractalkine and/or IL-15 do not significantly affect NK cell adhesion to MAdCAM-1, despite changes they elicit to the expression of integrin α4β7. This study provides further evidence that CX3CR1 antagonism has therapeutic utility in rescuing NK cells from the deleterious effects of the omentum and fractalkine in OAC, thus limiting their dysfunction.

## 1. Introduction

Oesophagogastric adenocarcinomas (OAC) are a group of obesity-associated malignancies which encompass oesophageal, gastric and gastro-oesophageal junctional adenocarcinomas. OAC is underpinned by severe immune dysregulation and inflammation [1,2,3]. The 5-year survival rates for oesophageal adenocarcinoma and gastric adenocarcinoma of 20% and 32%, respectively, are largely due to poor treatment response rates of less than 30%, meaning new therapeutic options are urgently required for a growing group of cancer patients [4,5,6,7,8].

The innate lymphocytes natural killer (NK) cells possess both cytotoxic and cytokine producing capabilities and play a key role in tumour immunosurveillance [9,10]. As such, NK cells are of great interest in the realm of cell-based immunotherapy. In the setting of OAC, our group have reported the enhanced recruitment of NK cells to the chemotactic cues from visceral adipose tissue (VAT), the largest depot of which is the omentum [11]. We have also reported that soluble factors within OAC omentum drive phenotypical and functional changes and induce apoptosis of NK cells [11,12]. Current immunotherapies available for OAC have an efficacy of less than 25%, probably due to the cold immunosuppressive tumour microenvironment which does not facilitate immunotherapy-mediated reinvigoration of the immune infiltrate of the tumour [13,14]. We propose that the misguided immune cell migration to OAC omentum compromises anti-tumour immunity and thus poses a significant challenge for immunotherapeutic efficacy in these patients.

Chemokines function as mediators of immune cell migration and inflammation, and have also been implicated as promotors of cancer metastasis, thus placing them as potential targets in an immunotherapeutic context [15,16]. Fractalkine (CX3CL1) is a multi-functional inflammatory chemokine which is abundant in the OAC omentum [17]. It has an established role in macrophage- and CD8^+^ T cell-mediated adipose tissue inflammation [17,18,19,20]. Fractalkine is unique in that it functions both in transmembrane and soluble forms and has been implicated in roles beyond immune cell migration including immune cell survival, cancer metastasis and chronic inflammation [21]. We have identified fractalkine as a key driver of erroneous NK and CD8^+^ T cell migration to the omentum in OAC and established its key role in shaping both the phenotype and function of NK cells [12,17]. As such, targeting the fractalkine: CX3CR1 pathway with therapeutic intent to reposition crucial T and NK cells from omentum to tumour is of great interest in OAC. However it is prudent to further explore the effects that fractalkine exerts on NK cell phenotype and function in OAC and unearth the biological consequences of targeting this pathway for therapeutic purposes.

IL-15 is an important NK cell cytokine and has generated considerable interest in the realm of cancer immunotherapy as an alternative to IL-2 [22]. Whilst IL-2 and IL-15 overlap in several key functions, including supporting the proliferation and activation of NK cells, IL-2 can support the persistence of regulatory T cells (Tregs), which is an undesirable feature in the context of anti-tumour immunity [23,24]. Here, we explore the impact of the OAC omentum microenvironment on NK cell migration to tumour in OAC and examine its influence on NK cell phenotype and function. As OAC omentum is a fractalkine- and IL-15-enriched tissue, we extend these studies to further investigate the role of fractalkine and IL-15 in this milieu.

For the first time, our data demonstrate fractalkine-mediated endocytosis of CX3CR1 in NK cells. Short term exposure to this chemokine is followed by recycling of CX3CR1 to the surface when NK cells are transferred to a fractalkine-free environment. Importantly, we report that exposure to the soluble factors of the omentum of OAC patients dampens NK cell migration towards the soluble chemotactic cues of OAC tumour. Furthermore, pre-treatment with a CX3CR1 antagonist can overcome this conditioning by the omental microenvironment and restore NK cell migration towards OAC tumour. Our data also reveal that exposure to soluble fractalkine significantly dampens IL-15-mediated stimulation of death receptor ligand and integrin expression on NK cells. Our novel study provides further evidence supporting the therapeutic utility of CX3CR1 antagonism to prevent NK cell trafficking to omentum and to limit the deleterious effects of fractalkine in viscerally obese OAC patients.

## 2. Materials and Methods

### 2.1. Patient Demographics

Blood, omentum and tumour tissues were collected from a total of 28 patients at time of surgical resection from patients attending the National Oesophageal and Gastric Centre at St James’s Hospital, Dublin. The group consisted of nine patients with confirmed oesophageal adenocarcinoma, 14 patients with oesophago-gastric junctional adenocarcinoma and five patients with gastric adenocarcinoma (Table 1). The group consisted of 20 males and eight females, representative of the predominance of OAC in males, with an average age 64 years. The mean BMI at the time of surgery was 29 kg/m^2^, making 85% of our cohort overweight or obese. The mean CT-defined VFA was 144 cm^2^. Neo-adjuvant chemoradiotherapy (CRT) was administered to 68% of patients.

The work was performed in accordance with the Code of Ethics of the World Medical Association (Declaration of Helsinki) for experiments involving humans. Patients provided informed consent for sample and data acquisition, and the study received full ethical approval from the St. James’s Hospital Ethics Review Board. Patient samples were pseudonymised to protect the privacy rights of the patients.

### 2.2. Sample Preparation

Blood, omental adipose tissue samples and tumour biopsies were collected during surgical resection. Five grams of omental adipose tissue was enzymatically digested with collagenase type II for 20 min to obtain the stromal vascular fraction (SVF) as previously described [25]. Tumour biopsies were enzymatically digested with collagenase type IV as previously described [26]. Adipose tissue conditioned media (ACM) and tumour tissue conditioned media (TCM) were prepared as previously described [12,27]. ACM derived from obese OAC patients was used in experiments to recapitulate the obese omental microenvironment as that is the predominant phenotype in OAC.

### 2.3. Patient Sample Phenotyping

Whole blood, SVF from omentum and intratumoural immune cells were stained with CD56-FITC-Viobright (Miltenyi Biotec, Bergisch Gladbach, Germany), L-selectin-BV510, CD3-APC-Cy7, Integrin β7-Pe-Cy5, α4-Pe-Cy7, TRAIL-APC, FasL-BV421 (Biolegend, San Diego, CA, USA). Red blood cells were lysed using BD Lysing Solution (BD Biosciences, Franklin Lakes, NJ, USA) as per manufacturer’s instructions. NK cells were quantified as CD56^+^CD3^-^ cells within the lymphocyte gate. Cells were acquired using the CANTO II (BD Biosciences) flow cytometer and analysed using FlowJo software (BD Biosciences).

### 2.4. CX3CR1 Endocytosis following Treatment with Recombinant Fractalkine

PBMC were isolated from non-cancer controls by density gradient centrifugation and seeded at a density of 1 × 10^6^ cells/mL RPMI supplemented with 10% foetal bovine serum (FBS) and 1% penicillin-streptomycin (cRPMI). To determine whether CX3CR1 was internalised via endocytosis following treatment with recombinant fractalkine, cells were treated with 30 ng/mL of recombinant fractalkine (Biolegend) alone or in the presence 80 µM of the dynamin inhibitor Dynasore (Sigma Aldrich, St. Louis, MO, USA) or cold treatment at 4 °C. Cells were stained for flow cytometric analysis with CD56-FITC-Viobright, CX3CR1-PE (Miltenyi Biotec) and CD3-APC-Cy7 (Biolegend). Cells were acquired using the CANTO II (BD Biosciences) flow cytometer and analysed using FlowJo software (BD Biosciences).

### 2.5. CX3CR1 Recycling following Treatment with Recombinant Fractalkine

PBMC were isolated from non-cancer controls by density gradient centrifugation and seeded at a density of 1 × 10^6^ cells/mL cRPMI. PBMC were treated with 100 ng/mL of recombinant fractalkine for 2 or 24 h (Peprotech, Cranbury, NJ, USA), subsequently washed and resuspended in fresh cRPMI for 24 and 48 h. Cells were stained for flow cytometric analysis with CD56-FITC-Viobright, CX3CR1-PE (Miltenyi Biotec, Bergisch Gladbach, Germany) and CD3-APC-Cy7 (Biolegend). Cells were acquired using the CANTO II (BD Biosciences) flow cytometer and analysed using FlowJo software (BD Biosciences).

### 2.6. Phenotyping NK Cells following Fractalkine and IL-15 Treatment

PBMC were isolated from non-cancer controls by density gradient centrifugation and seeded at a density of 1 × 10^6^ cells/mL cRPMI. Cells were treated with 30 ng/mL of recombinant fractalkine (Biolegend) and/or 100 ng/mL of recombinant IL-15 (Immunotools, Friesoythe, Germany) for 2 or 24 h. Cells were stained with CD56-FITC-Viobright (Miltenyi Biotec), L-selectin-BV510, CD3-APC-Cy7, Integrin β7-Pe-Cy5, α4-Pe-Cy7, TRAIL-APC, FasL-BV421 (Biolegend). Samples were acquired using the CANTO II (BD Biosciences) flow cytometer and analysed using FlowJo software (BD Biosciences).

### 2.7. OAC Patient-Derived Adipose Conditioned Media Treatment

PBMC were isolated from non-cancer controls by density gradient centrifugation and seeded at a density of 1 × 10^6^ cells/mL cRPMI. Cells were cultured with OAC patient- derived ACM for 2 and 24 h or M199. Cells were stained with CD56-FITC-Viobright (Miltenyi Biotec), L-selectin-BV510, CD3-APC-Cy7, Integrin β7-Pe-Cy5, α4-Pe-Cy7 (Biolegend). Samples were acquired using the CANTO II (BD Biosciences) flow cytometer and analysed using FlowJo software (BD Biosciences). 

### 2.8. NK Cell Adhesion Assay

Adhesion assay protocol was adapted from Strazza et al. [28]. Non-cancer control primary NK cells were isolated from PBMC by magnetic cell sorting using human NK cell isolation kit (Stemcell, Vancouver, British Columbia, Canada) according to manufacturer’s instructions. NK cells were resuspended at a density of 1 × 10^5^/100µL of NK MACS media (Miltenyi Biotec) supplemented with 100 IU IL-2 (Peprotech) and treated with 100 ng IL-15 (Immunotools) and/or 100 ng/mL fractalkine (Peprotech) for 2 and 24 h. NK cells were also treated with OAC patient-derived TCM or ACM for 2 and 24 h with and without pre-treatment with 5 nM CX3CR1 antagonist E6130 (MedChemExpress, Monmouth Junction, NJ, USA) for 1 h prior to exposure to ACM. A 96 well plate was coated with goat-anti-human IgG (Fc specific) in PBS and incubated overnight at 4 °C. Plates were subsequently coated with 2.5 µg/mL MAdCAM-1 (R&D, Minneapolis, MN, USA) for 1 h at 37 °C. NK cells were stained with CFSE and allowed to adhere for 20 min at 37 °C. Unbound cells were washed away. Unwashed wells were used as controls. The percentage of adherent cells was determined by a fluorescent plate reader and calculated as follows:

$$\frac{\mathrm{Avg}\;\mathrm{intensity}\;\mathrm{in}\;\mathrm{well}}{\mathrm{Avg}\;\mathrm{intensity}\;\mathrm{in}\;\mathrm{unwashed}\;\mathrm{well}}\;\times \;\frac{100}{1}$$

### 2.9. NK Cell Chemotaxis Assay

PBMC were isolated from non-cancer controls by density gradient centrifugation and resuspended in RPMI and treated with 100 ng fractalkine (Peprotech) and/or 100 ng IL-15 (Immunotools) for 2 or 24 h. Alternatively cells were treated with ACM for 2 or 24 h with and without pre-treatment with 5 nM of CX3CR1 antagonist E6130 (MedChemExpress) for 1 h before exposure to ACM. Cells were subsequently added at a density of 0.2 × 10^6^ cells/100 µL RPMI to a 5 µm pore Transwell filter system (Corning Inc, Corning, NY, USA) with TCM added in the lower chamber. M199 was used as a negative control and M199 supplemented with 20% FBS was used as a positive control. This system was incubated for 2 h at 37 °C, 5% CO_2_. Cells were collected from the lower chamber and stained for flow cytometric analysis with CD56-FITC-Viobright (Miltenyi Biotec) and CD3-APC-Cy7 (Biolegend). CountBright beads (ThermoFisher, Waltham, MA, United States) were used to enumerate the migrated CD56^+^CD3^-^ NK cells. Cells were acquired using the CANTO II (BD Biosciences) flow cytometer and analysed using FlowJo software (BD Biosciences).

### 2.10. Statistical Analysis

Statistical analysis was carried out using GraphPad Prism Version 8 (GraphPad Software, San Diego, CA, USA). Differences between groups were analysed using one-way ANOVA with Bonferroni post-hoc test or paired t-test where appropriate. *p* < 0.05 was considered significant.

## 3. Results

### 3.1. Fractalkine Mediates the Endocytosis of CX3CR1

**Figure 1:**
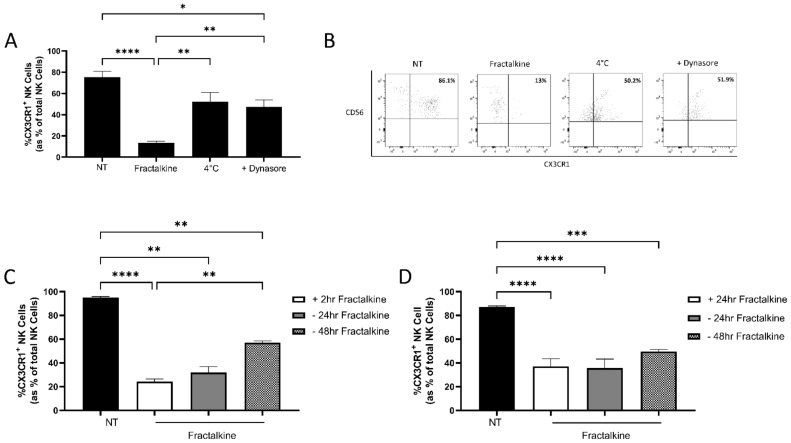
Fractalkine mediates endocytosis of CX3CR1 in NK cells. (**A**) Bar chart showing the frequencies of CX3CR1^+^ NK cells as a percentage of total NK cells following no treatment (NT), treatment with 30 ng/mL of recombinant fractalkine for 2 h alone, in combination with 80 µM of dynamin inhibitor Dynasore or at 4 °C. (**B**) Representative dot plots showing peripheral blood derived CX3CR1^+^ NK cells previously gated on total lymphocytes following no treatment (NT) or treatment with fractalkine for 2 h alone, at 4 °C or in combination with dynasore. (**C**) Bar chart showing the frequencies of CX3CR1^+^ NK cells as a percentage of total NK cells following no treatment (NT), treatment with fractalkine for 2 h (white) and following removal to a fractalkine-free environment for 24 h (grey) or 48 h (check). (**D**) Bar chart showing the frequencies of CX3CR1^+^ NK cells as a percentage of total NK cells following no treatment (NT), treatment with fractalkine for 24 h (white) and following removal to a fractalkine free environment for 24 h (grey) or 48 h (check). One way ANOVA, * *p* < 0.05, ** *p* < 0.01, *** *p* < 0.001, **** *p* < 0.0001.

To ascertain whether our previously reported fractalkine-mediated decreases in CX3CR1 expression by NK cells were due to receptor endocytosis, PBMC were treated with fractalkine along with the dynamin inhibitor dynasore or cold treatment at 4 °C (*n* = 9) [17]. Our data revealed significantly higher frequencies of CX3CR1^+^ NK cells following fractalkine treatment in combination with dynasore, or at 4 °C, compared to 2 hfractalkine treatment alone, suggesting reduced CX3CR1 expression is due to endocytosis; 2 h vs. dynasore (13.38% vs 47.36%, *p* = 0.0089), 2 h vs. cold treatment (13.38% vs 52.38%, *p* = 0.0099) (Figure 1A,B). It is of note that there is a loss of the CD56^BRIGHT^ population following treatment with fractalkine in combination with dynasore or at 4 °C. Our data strongly suggest that this is a result of the experimental conditions and not a fractalkine-mediated loss of CD56 expression. To ascertain whether removal to a fractalkine-free environment would restore CX3CR1 expression, NK cells were treated with fractalkine for 2 or 24 h and subsequently re-suspended in fractalkine-free media for 24 and 48 h. Following 2 h fractalkine treatment, subsequent resuspension in fractalkine-free media for 48 h significantly increased the frequencies of CX3CR1^+^ NK cells; 2 h vs. +48 h (35.9% vs. 57.1% *p* = 0.0070) (Figure 1C). There are however significantly less CX3CR1^+^ NK cells following 24 and 48 h removal to a fractalkine free environment and cells left untreated, suggesting the frequency CX3CR1^+^ is not fully restored; untreated vs. +24 h (95% vs. 32%, *p* = 0.0022), untreated vs. +48 h (90.9% vs. 57.1%, *p* = 0.0010). There were no significant differences in the frequency of CX3CR1^+^ NK cells treated for 24 h and those treated for 24 h and subsequently removed to a fractalkine free environment for 24 or 48 h. Furthermore, following 24 h fractalkine treatment and subsequent culture in a fractalkine-free environment for 24 and 48 h the frequencies of CX3CR1^+^ NK cells remained significantly lower than cells left untreated; untreated vs. +24 h (86% vs. 35.76%, *p* < 0.0001) untreated vs. +48 h (86% vs. 49.7%, *p* = 0.0004) (Figure 1D).

### 3.2. Exposure to ACM Significantly Reduces NK Cell Migration towards OAC Patient-Derived TCM

**Figure 2:**
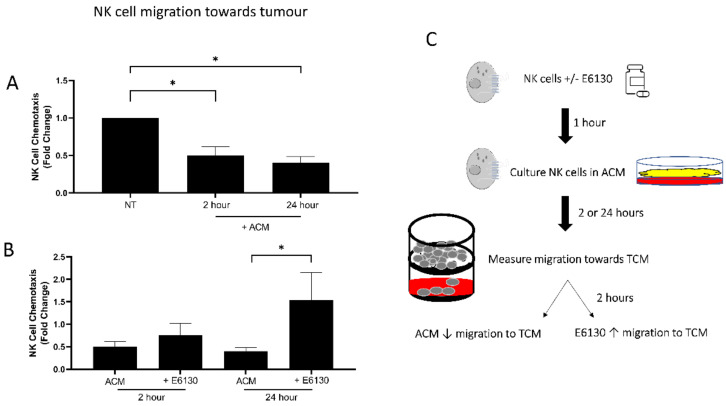
Soluble mediators in OAC patient-derived omentum significantly impede NK cell migration towards OAC tumour, which can be restored by CX3CR1 antagonist E6130. (**A**) Bar chart showing the fold-change migration of NK cells to OAC patient-derived tumour conditioned media (TCM) relative to no treatment (NT) or treatment with adipose conditioned media (+ACM) for 2 h or 24 h (*n* = 3–4). (**B**) Bar chart showing the fold-change migration of NK cells to OAC patient-derived tumour conditioned media (TCM) relative to no treatment, following treatment with adipose conditioned media (ACM) for 2 h or 24 h with and without pre-treatment with 5 nM CX3CR1 antagonist (+E6130) (*n* = 3–4). (**C**) Schematic of NK cell chemotaxis towards TCM. NK cells are pre-treated with CX3CR1 antagonist E6130 for 1 h prior to culture in ACM or left untreated. Cells are then cultured in OAC patient-derived ACM for 2 or 24 h. NK cell migration towards TCM is then measured in a transwell assay for 2 h. ACM exposure significantly decreases NK cell migration towards TCM, while pre-treatment significantly increases NK cell migration towards TCM. Paired *t*-test, * *p* < 0.05.

To elucidate the effects of the omental microenvironment on NK cell chemotaxis towards tumour, NK cells were exposed to obese OAC patient ACM with and without pre-treatment with CX3CR1 antagonist E6130. NK cell migration towards TCM was significantly reduced following exposure to ACM for 2 (*n* = 3) and 24 (*n* = 4) h; Untreated vs. 2 h (1 vs. 0.5026, *p* = 0.0229), untreated vs. 24 h (1 vs. 0.4019, *p* = 0.0185) (Figure 2A). Pre-treatment with a CX3CR1 antagonist for one hour prior to ACM exposure for 24 h significantly restored migration towards TCM compared to ACM alone (*n* = 3); 24 h ACM vs. 24 h ACM and CX3CR1 antagonist (0.4019 vs. 0.9345, *p* = 0.0114) (Figure 2B,C).

### 3.3. IL-15 Antagonises Fractalkine-Mediated Decreases in NK Cell Migration towards the Chemotactic Signals of OAC Tumour

**Figure 3:**
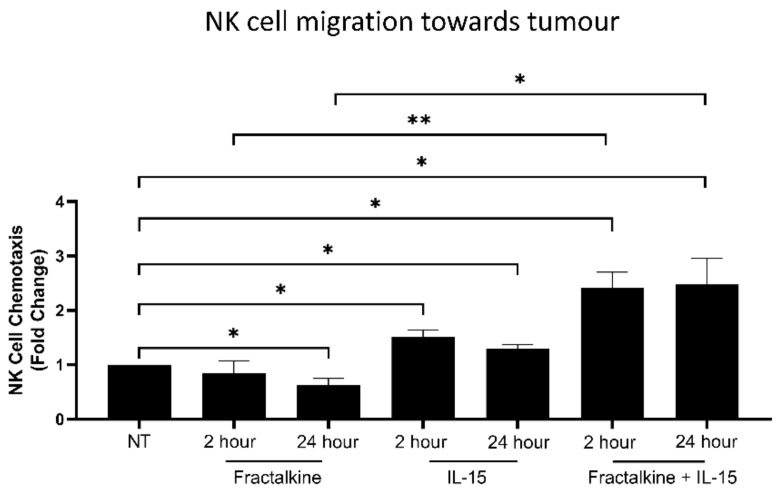
Fractalkine-induced impedance of NK cell migration to OAC tumour-derived conditioned media can be overcome by IL-15. Bar chart showing the fold-change migration of NK cells to OAC patient-derived TCM following no treatment, treatment with 100 ng/mL of fractalkine and/or IL-15 (*n* = 3–6). Paired t test, * *p* < 0.05, ** *p* < 0.01.

To elucidate the effects of fractalkine on NK cell migration, PBMC were treated with fractalkine for 2 or 24 h and migration towards TCM was measured with a transwell chemotaxis assay. Treatment with fractalkine for 24 h mediated a significant decrease in the migration of NK cell towards TCM compared to cells left untreated; untreated vs. 24 h fractalkine (1 vs. 0.63, *p* = 0.02) (Figure 3). Treatment with the well described NK cell activator IL-15 for 2 and 24 h significantly increased migration towards TCM; untreated vs. 2 h IL-15 (1 vs. 1.623, *p* = 0.0146), untreated vs. 24 h IL-15 (1 vs. 1.29, *p* = 0.0434) (Figure 3). Interestingly, treatment with both fractalkine and IL-15 for 2 and 24 h significantly increased migration towards TCM; untreated vs. 2 h IL-15 and fractalkine (1 vs. 2.41, *p* = 0.0166), untreated vs. 24 h IL-15 and fractalkine (1 vs. 2.47, *p* = 0.02). Furthermore, migration towards TCM was significantly increased following treatment with Fractalkine and IL-15 compared to fractalkine alone at 2 and 24 h; 2 h Fractalkine vs. Fractalkine and IL-15 (0.84 vs. 2.41, *p* = 0.0071), 24 h Fractalkine vs. Fractalkine and IL-15 (0.63 vs. 2.47, *p* = 0.0311) (Figure 3).

### 3.4. Fractalkine Antagonises IL-15-Mediated Increases in Adhesion Molecule Expression 

**Figure 4:**
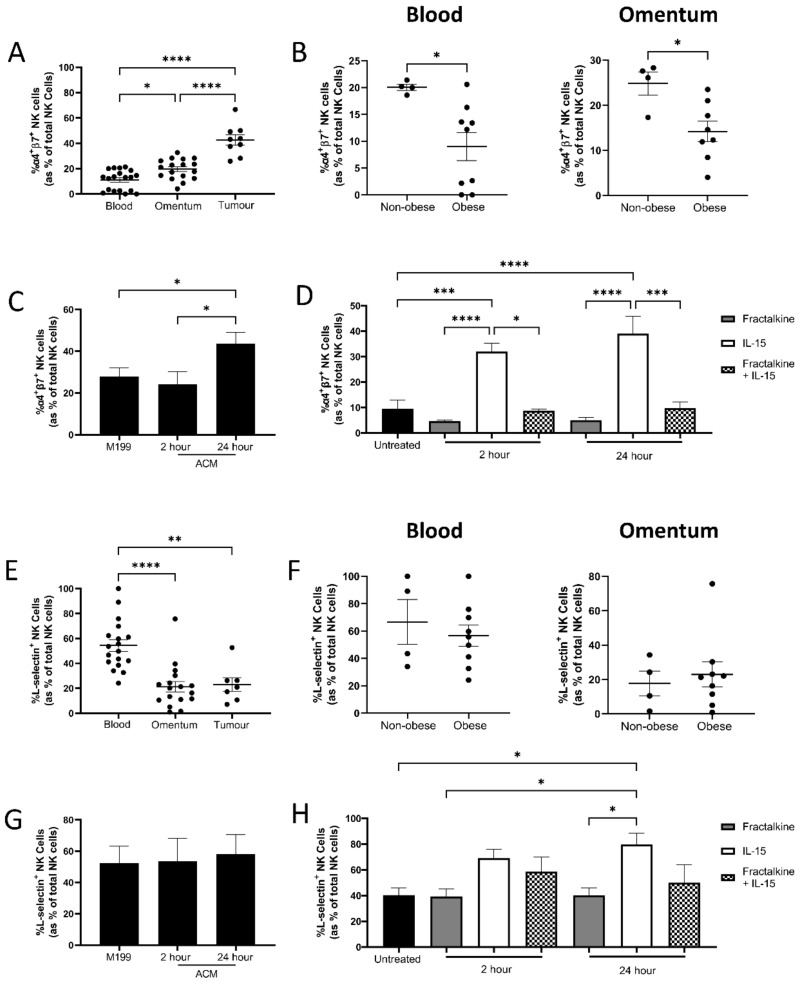
Fractalkine attenuates IL-15 induced expression of gut-homing integrins α4β7. (**A**) Dot plot showing the frequencies of α4^+^β7^+^ NK cells as a percentage of total NK cells in OAC patient derived blood (*n* = 18), omentum (*n* = 17) and tumour (*n* = 9). (**B**) Dot plot showing the frequencies of α4^+^β7^+^ NK cells as a percentage of total NK cells in OAC patient derived blood divided in to obese (*n* = 8-9) and non-obese (*n* = 4) by VFA in blood (left) and omentum (right). (**C)** Bar chart showing the frequencies of α4^+^β7^+^ NK cells following treatment with M199 or OAC patient derived adipose conditioned media for 2 and 24 h. (**D)** Bar chart showing the frequencies of α4^+^β7^+^ NK cells following no treatment (untreated) or treatment with 30 ng/mL fractalkine and/or 100 ng/mL IL-15 for 2 or 24 h (*n* = 3–14). (**E**) Dot plot showing the frequencies of L-selectin^+^ NK cells as a percentage of total NK cells in OAC patient derived blood (*n* = 18), omentum (*n* = 17) and tumour (*n* = 8). (**F**) Dot plot showing the frequencies of L-selectin^+^ NK cells as a percentage of total NK cells in OAC patient derived blood divided in to obese (*n* = 9) and non-obese (*n* = 4) by VFA in blood (left) and omentum (right). (**G**) Bar chart showing the frequencies of L-selectin^+^ NK cells following treatment with M199 or OAC patient derived adipose conditioned media for 2 and 24 h. (**H**) Bar chart showing the frequencies of L-selectin^+^ NK cells following no treatment (untreated) or treatment with 30 ng/mL fractalkine and/or 100 ng/mL IL-15 for 2 or 24 h (*n* = 3–14). One way ANOVA or *t*-test as appropriate, * *p* < 0.05, ** *p* < 0.01, *** *p* < 0.001, **** *p* < 0.0001.

To ascertain whether dysregulated NK cell migration was accompanied by altered adhesion in OAC, we first profiled integrin and adhesion molecule expression on NK cells from OAC patient-derived blood, omentum and tumour. There were significantly higher frequencies of NK cells expressing the gut-homing integrins α4 and β7 in the omentum (*n* = 17) and tumour (*n* = 9), compared to the circulation (*n* = 19); blood vs. omentum (11% vs. 19.6%, *p* = 0.0155), blood vs. tumour (11% vs. 42.6%, *p* < 0.0001). Similarly, there were significantly higher frequencies within the tumour of OAC patients, compared to the omentum; omentum vs. tumour (19.6% vs. 42.6%, *p* < 0.0001) (Figure 4A). There were significantly lower frequencies of NK cells expressing the adhesion molecule L-selectin in the omentum (*n* = 17) and tumour (*n* = 7) of OAC patients, compared to the circulation (*n* = 18); blood vs. omentum (54.43% vs. 21.24%, *p* < 0.0001), blood vs. tumour (54.43% vs.23.02%, *p* = 0.0012) (Figure 4E).

To examine the impact of obesity status on NK cell adhesion molecule expression, OAC patients were stratified into obese (*n* = 8–9) and non-obese (*n* = 4) by VFA. There were significantly lower frequencies of α4^+^β7^+^ NK cells in the circulation and omentum of obese OAC patients, compared to their non-obese counterparts; blood non-obese vs. obese (20.05% vs. 8.99%, *p* = 0.0188), omentum non-obese vs. obese (24.83% vs. 14.19%, *p* = 0.0169) (Figure 4B).

We next sought to determine whether soluble factors in the omentum alter NK cell adhesion in addition to migration. Blood-derived NK cells were treated with ACM for 2 or 24 h and then examined for integrin and adhesion molecule expression. There were significantly higher frequencies of the NK cells expressing the gut-homing integrins α4 and β7 following treatment with OAC ACM for 24 h compared to cells treated with M199 or ACM for 2 h; M199 vs. 24 h (27.98% vs. 43.7%, *p* = 0.04), 2 h vs. 24 h (24% vs. 43.7%, *p* = 0.03) (Figure 4c). There were no changes in the frequencies of cells expressing L-selectin following culture in ACM (Figure 4G).

Finally, blood-derived NK cells were treated with fractalkine and/or IL-15 for 2 or 24 h and then examined for integrin and adhesion molecule expression. Fractalkine alone had no effect on α4, β7 or L-selectin expression (Figure 4D,H). There were significantly higher frequencies of α4^+^β7^+^ NK cells following treatment with IL-15 for 2 and 24 h (*n* = 6) compared to cells left untreated (*n* = 14); untreated vs. 2 h (9.567% vs. 31.92%, *p* = 0.0001), untreated vs. 24 h (9.567% vs. 39.00%, *p* < 0.0001). Interestingly, there were significantly higher frequencies of α4^+^β7^+^ following stimulation with IL-15 compared with fractalkine alone for 2 and 24 h; 2 h IL-15 vs. fractalkine (31.92% vs. 4.6%, *p* < 0.0001), 24 h IL-15 vs. fractalkine (39% vs. 5.05%, *p* < 0.0001). Similarly, there were significantly higher frequencies of α4^+^β7^+^ NK cells following treatment with IL-15 compared with fractalkine and IL-15 at 2 and 24 h; 2 h IL-15 vs. fractalkine and IL-15 (31.92% vs. 8.78%, *p* = 0.0130), 24 h IL-15 vs. fractalkine and IL-15 (39% vs. 9.81%, *p* = 0.0008) (Figure 4D). There were significantly higher frequencies of L-selectin^+^ NK cells following treatment with IL-15 for 24 h (*n* = 3), compared to cells left untreated (*n* = 11); untreated vs. 24 h IL-15 (40.39% vs. 79.7%, *p* = 0.0420). However, there are no significant increases following treatment with fractalkine and IL-15, suggesting again that fractalkine antagonises IL-15-induced increases in L-selectin expression (Figure 4H).

### 3.5. The OAC Omental Microenvironment Significantly Decreases NK Cell Adhesion to MAdCAM-1

**Figure 5:**
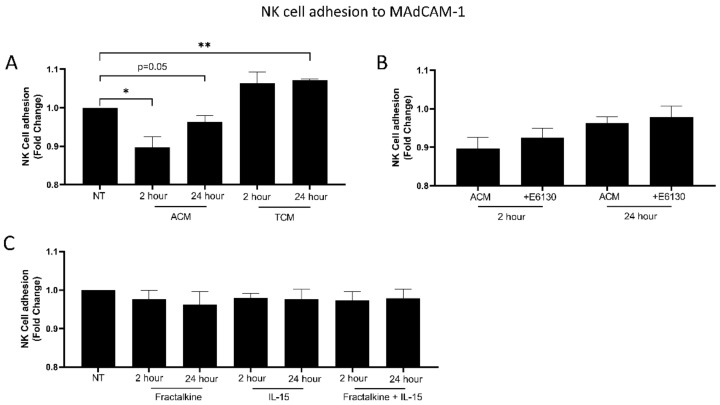
ACM slightly but significantly reduces NK cell adhesion to gut-homing adhesion molecule MAdCAM-1 whilst TCM significant increases it. (**A**) Bar chart showing the fold-change adhesion of NK cells to MAdCAM-1 following no treatment (NT), treatment with adipose conditioned media (ACM) or tumour conditioned media (TCM) for 2 or 24 h (*n* = 3–6). (**B**) Bar chart showing the fold-change adhesion of NK cells to MAdCAM-1 following treatment with adipose conditioned media (ACM) for 2 or 24 h with and without 1 h pre-treatment with 5 nM CX3CR1 antagonist E6130 (*n* = 3–6). (**C**) Bar chart showing the fold-change adhesion of NK cells to MAdCAM-1 following no treatment (NT), or treatment with 100 ng/mL of fractalkine and/or IL-15 (*n* = 3–6). Paired *t*-test, * *p* < 0.05, ** *p* < 0.01.

The effect of fractalkine and OAC patient-derived ACM on NK cell adhesion to MAdCAM-1 was determined via an adhesion assay. MAdCAM-1 is a gut-homing adhesion molecule known to play a key role in the trafficking of lymphocytes into inflamed tissues [29,30]. NK cell adhesion to MAdCAM-1 was significantly decreased following exposure to ACM for 2 (*n* = 5) and 24 h (*n* = 4) compared to cells left untreated; untreated vs 2 h ACM (1 vs. 0.89, *p*= 0.0215), untreated vs 24 h ACM (1 vs. 0.95, *p* = 0.05) (Figure 5A). There were no differences in NK cell adhesion when pre-treated with a CX3CR1 antagonist prior to exposure to ACM compared to cells not pre-treated (Figure 5B). NK cell adhesion to MAdCAM-1 was significantly increased following exposure to TCM for 24 h (*n* = 3) compared to cells left untreated; untreated vs 24 h TCM (1 vs. 1.07, *p* = 0.0021) (Figure 5A). There were no significant differences in adhesion following treatment with fractalkine, IL-15 or a combination of both (all *n* = 6) (Figure 5C).

### 3.6. Fractalkine Significantly Reduces IL-15-Mediated Stimulation of Death Receptor Ligand Expression by NK Cells

**Figure 6:**
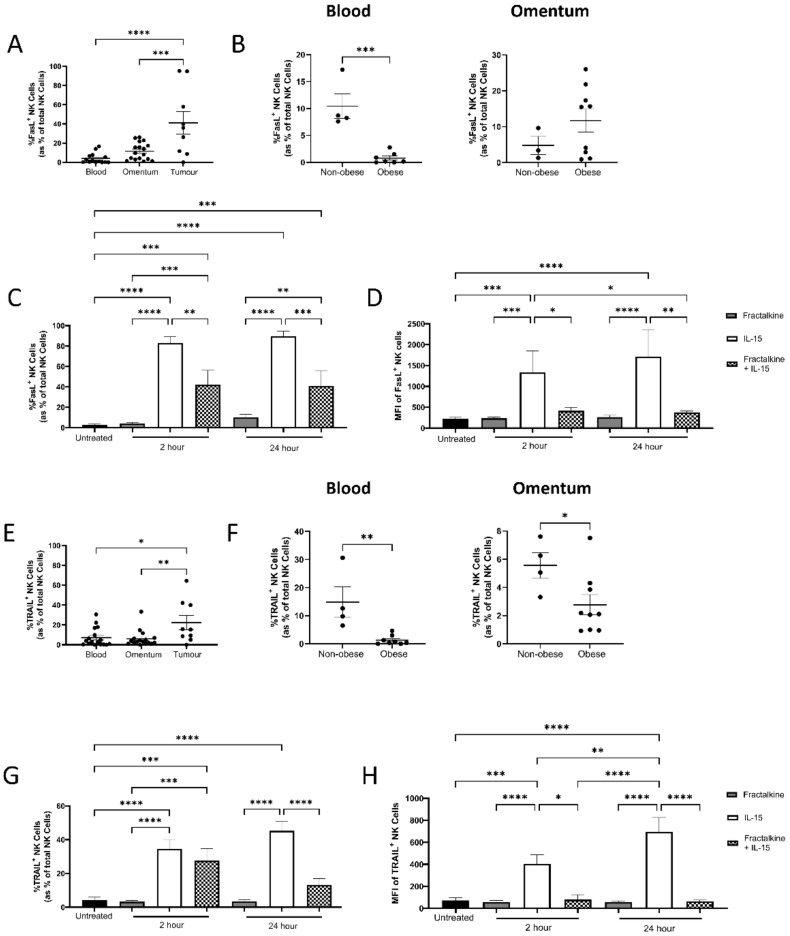
Death receptor ligand expressing NK cells are abundant in the OAC tumour and fractalkine antagonises the stimulatory effects of IL-15 on their expression. (**A**) Dot plot showing the frequencies of FasL ^+^ NK cells as a percentage of total NK cells in OAC patient derived blood (*n* = 14), omentum (*n* = 17) and tumour (*n* = 8). (**B**) Dot plot showing the frequencies of FasL^+^ NK cells as a percentage of total NK cells in OAC patient derived blood divided in to obese and non-obese by VFA in blood (left) and omentum (right). (**C**) Bar chart showing the frequencies of FasL^+^ NK cells as a percentage of total NK cells following no treatment (untreated), treatment with 30 ng/mL fractalkine and/or 100 ng/mL IL-15 for 2 and 24 h. (**D**) Bar chart showing the MFI of FasL^+^ expressing NK following no treatment (untreated), treatment with 30 ng/mL fractalkine and/or 100 ng/mL IL-15 for 2 and 24 h. (**E**) Dot plot showing the frequencies of TRAIL^+^ NK cells as a percentage of total NK cells in OAC patient derived blood (*n* = 20), omentum (*n* = 17) and tumour (*n* = 9). (**F**) Dot plot showing the frequencies of TRAIL^+^ NK cells as a percentage of total NK cells in OAC patient derived blood divided in to obese and non-obese by VFA in blood (left) and omentum (right). (**G**) Bar chart showing the frequencies of TRAIL^+^ NK cells as a percentage of total NK cells following no treatment (untreated), treatment with 30 ng/mL fractalkine and/or 100 ng/mL IL-15 for 2 and 24 h. (**H**) Bar chart showing the MFI of TRAIL expressing NK cells following no treatment (untreated), treatment with 30 ng/mL fractalkine and/or 100 ng/mL IL-15 for 2 and 24 h. One way ANOVA or *t*-test, * *p* < 0.05, ** *p* < 0.01, *** *p* < 0.001, **** *p* < 0.0001.

We next sought to profile death receptor ligand expression on NK cells from OAC patient blood, omentum and tumour. Death receptors are perforin and granzyme independent methods of NK cell cytotoxicity [31]. There were significantly higher frequencies of FasL^+^ NK cells in the tumour (*n* = 9) of OAC patients, compared to the circulation (*n* = 17) and omentum (*n* = 14); circulation vs. tumour (4.014% vs. 41.15%, *p* < 0.0001), omentum vs. tumour (11.44% vs. 41.15%, *p* = 0.0007) (Figure 6A). Similarly, there were significantly higher frequencies of TRAIL^+^ NK cells in the tumour (*n* = 9) of OAC patients, compared to the circulation (*n* = 17) and omentum (*n* = 17); circulation vs. tumour (7.143%vs. 22.32%, *p* = 0.0134), omentum vs. tumour (6.007% vs. 22.32%, *p* = 0.0075) (Figure 6E).

Interestingly, when patients were stratified into obese (*n* = 7) and non-obese (*n* = 4) cohorts by VFA, there were significantly lower frequencies of FasL^+^ NK cells in the circulation of the obese cohort compared to their non-obese counterparts; non-obese vs. obese (10.44% vs. 0.7943%, *p* = 0.0003) (Figure 6B). Similarly, there were significantly lower frequencies of TRAIL^+^ NK cells in the circulation of obese (*n* = 8) OAC patients compared to their non-obese (*n* = 4) counterparts; non-obese vs. obese (14.88% vs. 1.31%, *p* = 0.0044) (Figure 6F). Furthermore, in the omentum there were significantly lower frequencies of TRAIL^+^ NK cells in the omentum of obese (*n* = 9) OAC patients compared to their non-obese (*n* = 4) counterparts; non-obese vs. obese (5.563% vs. 2.769%, *p* = 0.0461) (Figure 6F).

The percentage frequencies of TRAIL^+^ and FasL^+^ NK cells were analysed following treatment with fractalkine and/or IL-15. There were significantly higher frequencies of NK cells expressing FasL following stimulation with IL-15 for 2 (*n* = 4) and 24 h (*n* = 3) compared to untreated cells (*n* = 11); untreated vs. 2 h (2.857% vs. 83.15%, *p* < 0.0001), untreated vs. 24 h (2.857% vs. 89.50%, *p* < 0.0001) (Figure 6B). Similar higher frequencies of TRAIL^+^ cells were observed following stimulation with IL-15 for 2 (*n* = 7) and 24 h (*n* = 6) compared to untreated cells (*n* = 11); untreated vs. 2 h (4.31% vs. 34.57%, *p* < 0.0001), untreated vs. 24 h (4.310% vs. 45.27%, *p* < 0.0001). Furthermore, there were significantly lower frequencies of FasL^+^ NK cells following treatment with fractalkine and IL-15 (*n* = 5) compared to cells treated with IL-15 alone (*n* = 6) for 2 and 24 h; 2 h IL-15 vs. fractalkine and IL-15 (83.15% vs. 42.09%, *p*= 0.0015), 24 h IL-15 vs. fractalkine and IL-15 (89.5% vs. 40.95%, *p*= 0.0009). Similarly, there were significantly lower frequencies of TRAIL^+^ NK cells following treatment with fractalkine and IL-15 (*n* = 5) compared to cells treated with IL-15 alone (*n* = 6) for 24 h; 24 h IL-15 vs. fractalkine and IL-15 (45% vs. 13%, *p* < 0.0001) (Figure 6B). 

To ascertain whether the expression levels of TRAIL and FasL on NK cells were altered by fractalkine and/or IL-15, the median fluorescence intensity (MFI) of their surface expression was analysed following treatment with fractalkine and/or IL-15. The MFI of FasL was significantly higher following stimulation with IL-15 for 2 and 24 h compared to untreated cells; untreated vs. 2 h (219 vs. 1334, *p* = 0.0008), untreated vs. 24 h (219 vs. 1715, *p* < 0.0001). Similarly, the MFI of TRAIL was significantly higher following stimulation with IL-15 for 2 and 24 h compared to untreated cells; untreated vs. 2 h (71.7 vs. 404, *p* = 0.0002), untreated vs. 24 h (71.7 vs. 695, *p* < 0.0001). Furthermore, the MFI of FasL was significantly lower following treatment with fractalkine and IL-15 compared to cells treated with IL-15 alone for 2 and 24 h; 2 h IL-15 vs. fractalkine and IL-15 (1334 vs. 415, *p* = 0.0475), 24 h IL-15 vs. fractalkine and IL-15 (1715 vs. 378, *p* = 0.0351). Similarly, the MFI of TRAIL was significantly lower following treatment with fractalkine and IL-15 compared to cells treated with IL-15 alone for 2 and 24 h; 2 h IL-15 vs. fractalkine and IL-15 (404 vs. 79, *p* = 0.0103)*,* 24 h IL-15 vs. fractalkine and IL-15 (695 vs. 63, *p* < 0.0001) (Figure 6C).

## 4. Discussion

Novel immunotherapeutic approaches, including those which utilise NK cells, are required to reinvigorate anti-tumour immunity and improve survival rates for OAC patients [4,5,6,7,8]. We propose that novel chemokine-based therapies which harness NK cell chemotactic pathways to boost migration to the solid OAC tumour may provide a means of augmenting anti-tumour immunity in OAC. Our group has reported the active recruitment of NK cells to OAC omentum and the alteration of NK cell viability, phenotype and function by soluble mediators within this microenvironment [11]. Furthermore we have identified the CX3CR1 ligand fractalkine as a key driver of NK cell migration to soluble factors in the OAC omentum, but not tumour, presenting CX3CR1 antagonism as an attractive therapeutic approach to impede erroneous NK cell recruitment to the omentum and concurrently, maximise their availability to traffic to the tumour in obese cancer patients [12].

Previous work by our group established fractalkine’s abundance within the omentum of OAC patients and demonstrated its capacity to significantly reduce the frequency of CX3CR1^+^ NK and CD8^+^ T cells in this compartment [12,17]. Here we report that this reduction is due to fractalkine-mediated endocytosis of CX3CR1 on the surface of NK cells. This is in line with previous work by our group which reported fractalkine-mediated endocytosis of CX3CR1 on the surface of CD8^+^ T cells [17]. Interestingly, and in contrast with results reported in relation to CD8^+^ T cells, short term treatment with fractalkine followed by subsequent removal to a fractalkine-free environment for 24 and 48 h allows for recycling of the receptor to the surface [17]. However, a longer 24 h treatment appears to impede this recycling. This suggests that the effects of fractalkine on NK cells is somewhat reversible and that preventing their long term exposure to high levels of fractalkine within the OAC omentum may allow for recycling of the receptor and restoration of the CX3CR1^HIGH^ NK cell phenotype [11]. In turn this may prevent the conversion of NK cell phenotype towards a CD27^+^ cytokine producing profile, which we propose is detrimental for anti-tumour immunity in OAC, and facilitate the maintenance of the cytotoxic CD27^-^ NK cell population [12].

Crucially and for the first time, we have uncovered that exposure to the soluble environment of the obese OAC omentum significantly reduces migration of NK cells towards OAC patient-derived TCM. This provides further evidence of the plethora of detrimental effects of visceral obesity on NK cell phenotype and function in OAC and provides further evidence that halting NK cell migration to the omentum is key to maximising their availability to infiltrate the tumour in OAC patients [11,12]. Importantly, pre-treatment with a CX3CR1 antagonist helps rescue NK cell chemotaxis towards TCM. This provides further evidence that fractalkine is a master regulator of NK cell migration, phenotype and function in OAC [12]. Furthermore, it adds merit to targeting the CX3CR1 pathway with therapeutic intent in OAC patients. It is well established that the infiltration of the solid tumour by NK cells is associated with improved prognosis, and as such, therapeutically boosting NK cell migration to OAC tumours is a desirable concept [32,33]. We have previously reported an abundance of CX3CR1^HIGH^ NK cells in the circulation of OAC patients, indicating their amenability to systemic administration of a CX3CR1 antagonist [12].

The cytokine IL-15 is being extensively explored in the setting of NK cell-based immunotherapies as it induces the proliferation and activation of NK cells [22,34]. Furthermore, IL-15 is a known promoter of NK cell migratory responses [35]. Since NK cells are exposed to both IL-15 and fractalkine in the omentum of OAC patients, we elucidated the effects of fractalkine on IL-15 stimulation of NK cell migration towards TCM. Interestingly, exposure to fractalkine for 24 h significantly decreases migration towards TCM, while as expected IL-15 enhances chemotaxis towards TCM. Importantly, the combination of IL-15 and fractalkine increases NK cell chemotaxis towards TCM. This further suggests IL-15 is a potent inducer of NK cell chemotaxis and can overcome fractalkine-mediated decreases in NK cell migration towards OAC tumours [35]. Moreover, it suggests that fractalkine and IL-15 synergise to promote NK cell migration and indicates that these two alone are not the soluble factors in OAC omentum which impede NK cell migration towards tumour in our ex vivo assays. This further supports our proposal to prevent circulating NK cells from migrating to the omentum and being exposed to the omental microenvironment, via the use of a CX3CR1 antagonist [12]. The adoptive transfer of immune cells, including NK cells, has had limited success in the setting of solid tumours and we have proposed that antagonism of specific chemokine receptors may prevent recruitment to the omentum and allow for homing of NK cells to the tumour in OAC [12,36]. As such, combination therapies including adoptive transfer of immune cells combined with antagonism of chemokine receptors to prevent migration towards the omentum could benefit from the addition of cytokines such as IL-15, which may serve to boost chemotaxis towards the tumour whilst also supporting and enhancing NK cell proliferation and cytotoxicity [22,34,35,37].

Since ACM obstructs NK cell migration towards tumour, we next sought to determine whether NK cells in OAC exhibit altered adhesion characteristics and if ACM promotes retention of NK cells within this tissue. Previous work by our group has reported altered adhesive capacities of CD8^+^ T cells following exposure to fractalkine, suggesting fractalkine may mediate cell retention in the omentum [17]. Firstly, we profiled the frequencies of NK cells expressing these adhesion molecules in our three compartments of interest, the blood, the omentum and the tumour. Here, we report significantly higher frequencies of NK cells expressing the gut-homing integrin α4β7 in the omentum and tumour of OAC patients, compared to the circulation. This is in line with previous work which has shown low levels of α4β7 expression of circulating NK cells [38]. α4β7 plays a key role in intestinal homing through its interactions with MAdCAM-1 [29,30]. MAdCAM-1 is expressed on the Peyer’s patches and mesenteric lymph nodes, along with the lamina propria of the intestines and its expression can be induced by TNF-α [39]. Importantly, the highly vascularised omentum contains vessels which express MAdCAM-1 and thus α4β7 is involved in migration of immune cells in to the omentum [30,40,41,42]. Increases in MAdCAM-1 expression have been reported in diseases underpinned by severe inflammation including inflammatory bowel disease, diabetes and cholangitis [30,43]. As such, the interactions between MAdCAM-1 and α4β7 are believed to play a critical role in facilitating the entry of lymphocytes into chronically inflamed tissue. As OAC is underpinned by inflammation, this suggests the increases in α4β7 expressing cells may be mediated by increased expression of MAdCAM-1 facilitating entry in to the inflamed omentum and tumour [44]. In support of our findings, exposure to OAC patient-derived ACM significantly increased the expression of α4β7 on NK cells, demonstrating that the omentum may be playing a role in shaping adhesion molecule expression in OAC.

To further explore the effects of fractalkine and IL-15 on NK cell migration and adhesion, the frequencies of cells expressing the adhesion molecules L-selectin and α4β7 were analysed. Interestingly, whilst IL-15 significantly increased the frequencies of cells expressing L-selectin and α4β7, simultaneous exposure to fractalkine undermines these effects, suggesting that fractalkine antagonises the stimulatory effects of IL-15 in relation to enhanced adhesion molecule expression. Previous work has shown short term stimulation with IL-15 increases L-selectin expression on NK cells, while a recent report has shown longer term stimulation can decrease L-selectin expression which can stifle NK cell expansion [45,46].

The highest proportion of L-selectin^+^ NK cells were identified in the circulation of OAC patients. L-selectin mediates the initial rolling step within the adhesion cascade and binds to MAdCAM-1 [47,48]. L-selectin is indispensable in the recruitment of NK cells to the tumour and the lymph nodes, allowing for NK cell tumour immunosurveillance [49,50]. Furthermore, L-selectin expression is indicative of a multi-functional NK cell with potent effector functions [51]. Our group has previously reported high levels of soluble L-selectin in the ACM of OAC patients, suggesting it may be shed by immune cells within this tissue and may influence immune cell retention within the omentum [17]. This suggests omental MAdCAM-1 may recruit L-selectin-expressing NK cells into the VAT and allow for their subsequent shedding of the ligand in this environment, thus contributing to the significantly lower frequencies seen within the omentum. Furthermore, we have previously identified a population of L-selectin^+^CX3CR1^+^ NK cells in the circulation of OAC patients, which we propose would be amenable to CX3CR1 antagonism and thus spared from recruitment to the omentum and subsequent loss of L-selectin expression [12].

Given the bias of gut-homing integrin expression in compartments of tumour and omentum, we next examined the adhesion of NK cells following exposure to these microenvironments. Adhesion to MAdCAM-1 was significantly increased following treatment with OAC patient-derived TCM. Adhesion is integral to the successful infiltration of lymphocytes into the solid tumour, with so called “cold” tumours often displaying disordered adhesion molecule expression on tumour vessels, thus leading to poorer infiltration [52]. This suggests the OAC tumour microenvironment leads to enhanced adhesion, which may facilitate the successful infiltration of immune cells into the solid tumour once they reach this site. In contrast, adhesion to MAdCAM-1 was mildly but significantly lessened following exposure to the ACM suggesting that this is not as important for adhesion within the omentum. Interestingly, pre-treatment with a CX3CR1 antagonist did not dampen this, suggesting that while antagonism can rescue ACM mediated alternations in NK cell migration, it cannot alter the adhesive capacity of NK cells.

In light of changes in α4β7 and L-selectin expression on NK cells observed following IL-15 treatment, we next wanted to determine whether treatment with fractalkine and IL-15 can alter NK cell adhesion to the ligand of α4β7 and L-selectin, MAdCAM-1. However, no differences in NK cell adhesion to MAdCAM-1 were observed following treatment with fractalkine, IL-15 or both. IL-15 is known to induce NK cell adhesion to endothelial cells, however these results suggest adhesion to MAdCAM-1 is not impacted [35].

Our data show that exposure to soluble factors in the OAC omental microenvironment, namely fractalkine, can significantly obstruct passage of NK cells to the tumour. This places more emphasis on finding a therapeutic means to prevent NK cell trafficking to the omentum in obesity-associated cancer and our data suggest that CX3CR1 antagonism provides the solution. We next sought to examine whether exposure to the omental secreted factors compromised the killing potential of NK cells via modulation of their death receptor ligands. Death receptor ligands such as TRAIL and FasL provide an alternative mode for NK cell cytotoxicity [31,53,54]. TRAIL is expressed at low levels on circulating NK cells but can be induced by cytokine stimulation [55]. FasL, the cognate ligand of Fas is expressed on cytotoxic effector cells, such as NK cells [56]. Our data show that the frequencies of TRAIL and FasL-expressing NK cells are significantly higher within the tumour of OAC patients, compared to the blood and omentum, indicating that once NK cells infiltrate the tumour microenvironment (TME), they are not inhibited from eliciting their killing activities. These data suggest that therapeutically boosting NK cell infiltration of OAC tumour may lead to enhanced killing. Overall, this suggests that the OAC TME is not completely immunosuppressive and that NK cells which successfully infiltrate the OAC tumour may have cytotoxic potential. Interestingly, there were significantly lower frequencies of TRAIL^+^ and FasL^+^ NK cells in the circulation of obese OAC patients compared to their non-obese counterparts. This is indicative of the often dysfunctional state of NK cells in obesity and is in agreement with a previous report which showed diminished TRAIL and CD107a expression on circulating NK cells from obese patients [57,58,59]. Importantly, treatment with fractalkine impedes IL-15-stimulated NK cell expression of TRAIL and FasL and presents further rationale to target this pathway via CX3CR1 antagonism. Overall these data suggest that entry into the fractalkine-rich omentum may not only compromise subsequent migration to the tumour but also attenuate the cytotoxic capacity of NK cells.

## 5. Conclusions

**Figure 7:**
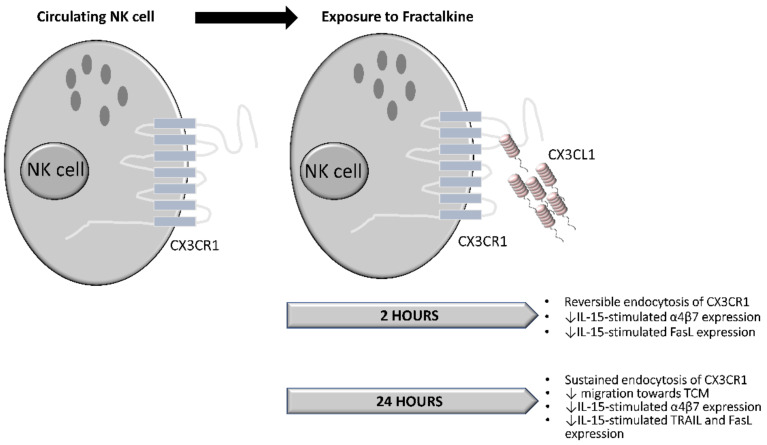
NK cell fate following exposure to fractalkine. Circulating NK cells are exposed to fractalkine. After 2 h of exposure there is reversible endocytosis of CX3CR1. When NK cells are stimulated with IL-15, fractalkine diminishes IL-15-mediated increases in α4β7 and FasL expression. After 24 h of exposure endocytosis of CX3CR1 is sustained. Migration towards TCM is decreased. Furthermore, IL-15-mediated increases in α4β7, TRAIL and FasL expression are dampened by fractalkine.

Here, we report the endocytosis of CX3CR1 on the surface of NK cells is reversible following short-term exposure to fractalkine suggesting that NK cell phenotype can be rescued in OAC (Figure 7). Crucially, we report for the first time that exposure to the soluble mediators of the OAC omentum significantly dampens NK cell migration towards OAC patient tumour providing further rationale to limit their recruitment to the VAT. Importantly, pre-treatment with a CX3CR1 antagonist can help rescue this migration, further indicating the central role that fractalkine plays in NK cell phenotype, function and migration in obesity-associated cancer. Furthermore, exposure to soluble fractalkine can dampen IL-15-mediated stimulatory effects on NK cell cytotoxicity markers and gut-homing integrins. Overall, this study presents further evidence that CX3CR1 antagonism holds therapeutic potential to rescue NK cells from fractalkine-mediated recruitment and subsequent alterations in omentum. In addition, our data provide novel insights into the utility of combining CX3CR1 antagonism with IL-15 to boost NK cell tumour-homing and tumour-killing in OAC patients.

## Figures and Tables

**Table 1:** Patient Demographics.

Age (years)	64
Sex ratio (M:F)	20:8
Diagnosis (no. patients)	
OAC	9
OGJ	14
Gastric	5
Tumour Stage ^a^ (no. patients)	
T0	3
T1	6
T2	7
T3	6
T4	5
Nodal Status ^b^ (no. patients)	
Positive	12
Negative	13
Mean BMI (kg/m^2^)	29
BMI ^c^ (no. patients)	
Underweight (BMI < 19.9)	0
Normal Weight (BMI 20–24.9)	4
Overweight (BMI 25–29.9)	12
Obese (BMI > 30)	11
Mean VFA (cm^2^) ^d^	144
Viscerally Obese by VFA ^e^	60%
Received Neoadjuvant CRT	68%

^a^ Tumour stage was unavailable for one patient. ^b^ Nodal status was unavailable for one patient. ^c^ BMI was unavailable for one patient. ^d^ VFA was unavailable for 10 patients. ^e^ Obese visceral fat area (VFA) > 160 cm^2^ for men and >80 cm^2^ for women (Doyle et al. 2013).

## Data Availability

Data available on request.

## References

[1] Lauby-Secretan B., Scoccianti C., Loomis D., Grosse Y., Bianchini F., Straif K. (2016). Body Fatness and Cancer—Viewpoint of the IARC Working Group. N. Engl. J. Med..

[2] Hoyo C., Cook M.B., Kamangar F., Freedman N.D., Whiteman D.C., Bernstein L., Brown L.M., Risch H.A., Ye W., Sharp L. (2012). Body mass index in relation to oesophageal and oesophagogastric junction adenocarcinomas: A pooled analysis from the international BEACON consortium. Int. J. Epidemiol..

[3] O’Sullivan K.E., Phelan J.J., O’Hanlon C., Lysaght J., O’Sullivan J.N., Reynolds J.V. (2014). The role of inflammation in cancer of the esophagus. Expert Rev. Gastroenterol. Hepatol..

[4] Siegel R.L., Miller K.D., Jemal A. (2020). Cancer statistics, 2020. CA. Cancer J. Clin..

[5] Van Hagen P., Hulshof M.C.C.M., Van Lanschot J.J.B., Steyerberg E.W., Henegouwen M.V.B., Wijnhoven B.P.L., Richel D.J., Nieuwenhuijzen G.A.P., Hospers G.A.P., Bonenkamp J.J. (2012). Preoperative Chemoradiotherapy for Esophageal or Junctional Cancer. N. Engl. J. Med..

[6] (2021). ASCO, Stomach Cancer: Statistics, Cancer.Net. www.cancer.net/cancer-types/stomach-cancer/statistics.

[7] Ajani J.A., Mansfield P.F., Janjan N., Morris J., Pisters P.W., Lynch P.M., Feig B., Myerson R., Nivers R., Cohen D.S. (2004). Multi-institutional trial of preoperative chemoradiotherapy in patients with potentially resectable gastric carcinoma. J. Clin. Oncol..

[8] Ajani J.A., Mansfield P.F., Crane C.H., Wu T.T., Lunagomez S., Lynch P.M., Janjan N., Feig B., Faust J., Yao J.C. (2005). Paclitaxel-Based Chemoradiotherapy in Localized Gastric Carcinoma: Degree of Pathologic Response and Not Clinical Parameters Dictated Patient Outcome. J. Clin. Oncol..

[9] Vivier E., Tomasello E., Baratin M., Walzer T., Ugolini S. (2008). Functions of natural killer cells. Nat. Immunol..

[10] Waldhauer I., Steinle A. (2008). NK cells and cancer immunosurveillance. Oncogene.

[11] Conroy M.J., Fitzgerald V., Doyle S.L., Channon S., Useckaite Z., Gilmartin N., O’Farrelly C., Ravi N., Reynolds J.V., Lysaght J. (2016). The microenvironment of visceral adipose tissue and liver alter natural killer cell viability and function. J. Leukoc. Biol..

[12] Mylod E., Melo A.M., Donlon N.E., Davern M., Bhardwaj A., Reynolds J.V., Lysaght J., Conroy M.J. (2021). Fractalkine Elicits Chemotactic, Phenotypic, and Functional Effects on CX3CR1 + CD27 − NK Cells in Obesity-Associated Cancer. J. Immunol..

[13] Lin E.W., Karakasheva T.A., Hicks P.D., Bass A.J., Rustgi A.K. (2016). The tumor microenvironment in esophageal cancer. Oncogene.

[14] Muro K., Chung H.C., Shankaran V., Geva R., Catenacci D., Gupta S., Eder J.P., Golan T., Le D.T., Burtness B. (2016). Pembrolizumab for patients with PD-L1-positive advanced gastric cancer (KEYNOTE-012): A multicentre, open-label, phase 1b trial. Lancet Oncol..

[15] Charo I.F., Ransohoff R.M. (2006). Mechanisms of disease: The many roles of chemokines and chemokine receptors in inflammation. N. Engl. J. Med..

[16] O’Donovan C., Davern M., Donlon N.E., Lysaght J., Conroy M.J. (2021). Chemokine-targeted therapies: An opportunity to remodel immune profiles in gastro-oesophageal tumours. Cancer Lett..

[17] Conroy M.J., Maher S.G., Melo A.M., Doyle S.L., Foley E., Reynolds J.V., Long A., Lysaght J. (2018). Identifying a novel role for fractalkine (CX3CL1) in memory CD8+ T cell accumulation in the omentum of obesity-associated cancer patients. Front. Immunol..

[18] Polyák A., Ferenczi S., Dénes A., Winkler Z., Kriszt R., Pintér-Kübler B., Kovács K.J. (2014). The fractalkine/Cx3CR1 system is implicated in the development of metabolic visceral adipose tissue inflammation in obesity. Brain Behav Immun..

[19] Shah R., Hinkle C.C., Ferguson J.F., Mehta N.N., Li M., Qu L., Lu Y., Putt M.E., Ahima R.S., Reilly M.P. (2011). Fractalkine is a novel human adipochemokine associated with type 2 diabetes. Diabetes.

[20] D’Haese J.G., Demir I.E., Friess H., Ceyhan G.O. (2010). Fractalkine/CX3CR1: Why a single chemokine-receptor duo bears a major and unique therapeutic potential. Expert Opin. Ther. Targets.

[21] Conroy M.J., Lysaght J. (2020). CX3CL1 Signaling in the Tumor Microenvironment. Adv. Exp. Med. Biol..

[22] Waldmann T.A. (2014). Interleukin-15 in the treatment of cancer. Expert Rev. Clin. Immunol..

[23] Waldmann T.A. (2006). The biology of interleukin-2 and interleukin-15: Implications for cancer therapy and vaccine design. Nat. Rev. Immunol..

[24] Yang Y., Lundqvist A. (2020). Immunomodulatory Effects of IL-2 and IL-15; Implications for Cancer Immunotherapy. Cancers.

[25] Lysaght J., Allott E.H., Donohoe C.L., Howard J.M., Pidgeon G.P., Reynolds J.V. (2011). T lymphocyte activation in visceral adipose tissue of patients with oesophageal adenocarcinoma. Br. J. Surg..

[26] Melo A.M., O’Brien A.M., Phelan J.J., Kennedy S.A., Wood N.A.W., Veerapen N., Besra G.S., Clarke N.E., Foley E.K., Ravi A. (2019). Mucosal-Associated Invariant T Cells Display Diminished Effector Capacity in Oesophageal Adenocarcinoma. Front. Immunol..

[27] Lysaght J., van der Stok E.P., Allott E.H., Casey R., Donohoe C.L., Howard J.M., McGarrigle S.A., Ravi N., Reynolds J.V., Pidgeon G.P. (2011). Pro-inflammatory and tumour proliferative properties of excess visceral adipose tissue. Cancer Lett..

[28] Strazza M., Azoulay-Alfaguter I., Pedoeem A., Mor A. (2014). Static Adhesion Assay for the Study of Integrin Activation in T Lymphocytes. J. Vis. Exp..

[29] Wagner N., Löhler J., Kunkel E.J., Ley K., Leung E., Krissansen G., Rajewsky K., Müller W. (1996). Critical role for beta7 integrins in formation of the gut-associated lymphoid tissue. Nature.

[30] Briskin M., Winsor-Hines D., Shyjan A., Cochran N., Bloom S., Wilson J., McEvoy L.M., Butcher E.C., Kassam N., Mackay C.R. (1997). Human mucosal addressin cell adhesion molecule-1 is preferentially expressed in intestinal tract and associated lymphoid tissue. Am. J. Pathol..

[31] Smyth M.J., Cretney E., Kelly J.M., Westwood J.A., Street S.E.A., Yagita H., Takeda K., Dommelen S.L.H.V., Degli-Esposti M.A., Hayakawa Y. (2005). Activation of NK cell cytotoxicity. Mol. Immunol..

[32] Ishigami S., Natsugoe S., Tokuda K., Nakajo A., Che X., Iwashige H., Aridome K., Hokita S., Aikou T. (2000). Prognostic value of intratumoral natural killer cells in gastric carcinoma. Cancer.

[33] Xu B., Chen L., Li J., Zheng X., Shi L., Wu C., Jiang J. (2016). Prognostic value of tumor infiltrating NK cells and macrophages in stage II+III esophageal cancer patients. Oncotarget.

[34] Berger C., Berger M., Hackman R.C., Gough M., Elliott C., Jensen M.C., Riddell S.R. (2009). Safety and immunologic effects of IL-15 administration in nonhuman primates. Blood.

[35] Allavena P., Giardina G., Bianchi G., Mantovani A. (1997). IL-15 is chemotactic for natural killer cells and stimulates their adhesion to vascular endothelium. J. Leukoc. Biol..

[36] Nayyar G., Chu Y., Cairo M.S. (2019). Overcoming resistance to natural killer cell based immunotherapies for solid tumors. Front. Oncol..

[37] Zhang C., Zhang J., Niu J., Zhang J., Tian Z. (2008). Interleukin-15 improves cytotoxicity of natural killer cells via up-regulating NKG2D and cytotoxic effector molecule expression as well as STAT1 and ERK1/2 phosphorylation. Cytokine.

[38] Pérez-Villar J.J., Zapata J.M., Melero I., Postigo A., Sánchez-Madrid E., López-Botet M. (1996). Expression and function of alpha 4/beta 7 integrin on human natural killer cells. Immunology.

[39] Connor E.M., Eppihimer M.J., Morise Z., Granger D.N., Grisham M.B. (1999). Expression of mucosal addressin cell adhesion molecule-1 (MAdCAM-1) in acute and chronic inflammation. J. Leukoc. Biol..

[40] Berberich S., Dähne S., Schippers A., Peters T., Müller W., Kremmer E., Förster R., Pabst O. (2008). Differential molecular and anatomical basis for B cell migration into the peritoneal cavity and omental milky spots. J. Immunol..

[41] Carlow D.A., Gold M.R., Ziltener H.J. (2009). Lymphocytes in the peritoneum home to the omentum and are activated by resident dendritic cells. J. Immunol..

[42] Meza-Perez S., Randall T.D. (2017). Immunological Functions of the Omentum. Trends Immunol..

[43] Salmi M., Andrew D.P., Butcher E.C., Jalkanen S. (1995). Dual binding capacity of mucosal immunoblasts to mucosal and synovial endothelium in humans: Dissection of the molecular mechanisms. J. Exp. Med..

[44] Kavanagh M.E., Conroy M.J., Clarke N.E., Gilmartin N.T., O’Sullivan K.E., Feighery R., MacCarthy F., O’Toole D., Ravi N., Reynolds J.V. (2016). Impact of the inflammatory microenvironment on T-cell phenotype in the progression from reflux oesophagitis to Barrett oesophagus and oesophageal adenocarcinoma. Cancer Lett..

[45] Lin S.-J., Chen J.-Y., Kuo M.-L., Hsiao H.-S., Lee P.-T., Huang J.-L. (2016). Effect of Interleukin-15 on CD11b, CD54, and CD62L Expression on Natural Killer Cell and Natural Killer T-Like Cells in Systemic Lupus Erythematosus. Mediators Inflamm..

[46] Mishra H.K., Dixon K.J., Pore N., Felices M., Miller J.S., Walcheck B. (2021). Activation of ADAM17 by IL-15 Limits Human NK Cell Proliferation. Front. Immunol..

[47] Yamada M., Yanaba K., Hasegawa M., Matsushita Y., Horikawa M., Komura K., Matsushita T., Kawasuji A., Fujita T., Takehara K. (2006). Regulation of local and metastatic host-mediated anti-tumour mechanisms by l-selectin and intercellular adhesion molecule-1. Clin. Exp. Immunol..

[48] Berg E.L., McEvoy L.M., Berlin C., Bargatze R.F., Butcher E.C. (1993). L-selectin-mediated lymphocyte rolling on MAdCAM-1. Nature.

[49] Sobolev O., Stern P., Lacy-Hulbert A., Hynes R.O. (2009). Natural killer cells require selectins for suppression of subcutaneous tumors. Cancer Res..

[50] Chen S., Kawashima H., Lowe J.B., Lanier L.L., Fukuda M. (2005). Suppression of tumor formation in lymph nodes by L-selectin-mediated natural killer cell recruitment. J. Exp. Med..

[51] Juelke K., Killig M., Luetke-Eversloh M., Parente E., Gruen J., Morandi B., Ferlazzo G., Thiel A., Schmitt-Knosalla I., Romagnani C. (2010). CD62L expression identifies a unique subset of polyfunctional CD56 dim NK cells. Blood.

[52] Harjunpää H., Llort Asens M., Guenther C., Fagerholm S.C. (2019). Cell Adhesion Molecules and Their Roles and Regulation in the Immune and Tumor Microenvironment. Front. Immunol..

[53] Cibrián D., Sánchez-Madrid F. (2017). CD69: From activation marker to metabolic gatekeeper. Eur. J. Immunol..

[54] Zamai L., Ahmad M., Bennett I.M., Azzoni L., Alnemri E.S., Perussia B. (1998). Natural Killer (NK) Cell–mediated Cytotoxicity: Differential Use of TRAIL and Fas Ligand by Immature and Mature Primary Human NK Cells. J. Exp. Med..

[55] Mirandola P., Ponti C., Gobbi G., Sponzilli I., Vaccarezza M., Cocco L., Zauli G., Secchiero P., Manzoli F.A., Vitale M. (2004). Activated human NK and CD8+ T cells express both TNF-related apoptosis-inducing ligand (TRAIL) and TRAIL receptors but are resistant to TRAIL-mediated cytotoxicity. Blood.

[56] Peter M.E., Hadji A., Murmann A.E., Brockway S., Putzbach W., Pattanayak A., Ceppi P. (2015). The role of CD95 and CD95 ligand in cancer. Cell Death Differ..

[57] O’Shea D., Hogan A.E. (2019). Dysregulation of Natural Killer Cells in Obesity. Cancers.

[58] Bähr I., Spielmann J., Quandt D., Kielstein H. (2020). Obesity-Associated Alterations of Natural Killer Cells and Immunosurveillance of Cancer. Front. Immunol..

[59] Laue T., Wrann C.D., Hoffmann-Castendiek B., Pietsch D., Hübner L., Kielstein H. (2015). Altered NK cell function in obese healthy humans. BMC Obes..

